# A Validation Study of a Polymer Optical Fiber Sensor for Monitoring Lumbar Spine Movement

**DOI:** 10.3390/ma12050762

**Published:** 2019-03-06

**Authors:** Wern Kam, Mary O’Keeffe, Kieran O’Sullivan, Waleed S. Mohammed, Sinead O’Keeffe, Elfed Lewis, Charusluk Viphavakit

**Affiliations:** 1 Optical Fibre Sensors Research Centre (OFSRC), Dept. of Electronic and Computer Engineering, University of Limerick, Limerick V94 T9PX, Ireland; joejoe_89@hotmail.com (W.K.); Sinead.OKeeffe@ul.ie (S.O.); elfed.lewis@ul.ie (E.L.); 2 School of Allied Health, University of Limerick, Limerick V94 T9PX, Ireland; mary.okeeffe@sydney.edu.au (M.O.); Kieran.OSullivan@aspetar.com (K.O.); 3 School of Public Health, University of Sydney, Sydney, NSW 2006, Australia; 4 Sports Spine Centre, Aspetar Orthopaedic and Sports Medicine Hospital, Doha 29222, Qatar; 5 Health Research Institute, University of Limerick, Limerick V94 T9PX, Ireland; 6 Center of Research in Optoelectronics, Communication and Control Systems (BU-CROCCS), School of Engineering, Bangkok University, Pathumthani 12120, Thailand; wsoliman@gmail.com; 7 International School of Engineering (ISE), Faculty of Engineering, Chulalongkorn University, Pathumwan, Bangkok 10330, Thailand

**Keywords:** plastic optical fibre sensor, range of motion, lumbar spine movement

## Abstract

This study aims to investigate the validity and reliability of a novel plastic optical fiber (POF) sensor, which was developed to measure the angles of flexion, extension and lateral bend at the lumbar region. The angles of flexion, extension and lateral bend for a standing position were measured simultaneously using both the novel POF sensor of this investigation and the commercial Biometrics goniometer instrument. Each movement had two steps of bending which were 10° and 20° based on inclinometer readings. The POF sensor had good intra-rater reliability (Intraclass correlation coefficient, ICC = 0.61 to 0.83). Bland–Altman plots were used to study the agreement using these two sensors. There were proportional differences and bias between the POF sensor and Biometrics goniometer, as the zero points did not lie in the percentage difference region in the Bland–Altman plots. The proportional difference between these two likely reflects the different sizes and thus, measurement regions of the two sensors. There was also strong correlation between the two sensors (r > 0.77). Hence, the POF sensor could be of potential utility in measuring lumbar range of motion (ROM) in a manner which is minimally invasive, and where discrete sections of the spine are under specific investigation.

## 1. Introduction

Low back pain (LBP) is a very common health problem and a leading cause of disability worldwide [1]. LBP can develop in any person of any age. There are a range of factors associated with LBP which include increasing age, lack of exercise, obesity, smoking, depression and workplace factors [2,3,4,5].

Low back movement can be analyzed using surface markers in laboratories which is the most common method [6]. However, it does not provide a highly accurate measurement compared to radiographic imaging [7]. Radiographic imaging includes radiography scanning modalities, e.g., X-rays [8] and magnetic resonance imaging (MRI) [9], as well as computer assisted tomography (CAT) scanning [10]. These methods provide an accurate measurement without any direct contact to human skin. However, the radiation exposed from the use of ionizing radiation in the measurement process can pose potential risks to a patient’s health [11]. In addition, the measurement processes are time-consuming, and their capital and maintenance costs are very high.

Other methods of analyzing low back movement include devices based on accelerometers [12,13], strain gauge sensors [14], inclinometers [15], gyroscopes [16] and the goniometer [17]. These devices can provide a static spinal posture of the patients. However, none of them provide a dynamic posture or dynamic angle of flexion [18,19]. In addition, the size of most of these classes of sensors are too bulky to wear, and they cannot localize motion analysis to specific sections of the spine. Previous work [6] has shown that the motion of the lumbar spine differs between upper and lower lumbar regions. A plastic optical fiber (POF) sensor developed by Kam et al. [20] is proposed as an alternative device to measure the spinal posture including the flexion angles at the low back region. The POF sensor has been designed as a highly compact and minimally intrusive measuring device. It is 7 cm long and 2.5 cm wide with a 3 cm gap between two attaching pods, which can be used to measure at discrete sections of the lumbar spine. It provides a dynamic measurement of the flexion angles with excellent time resolution. In this paper, the POF sensor is analyzed for its validity by comparing the measurements directly with a commercial Biometrics goniometer instrument in terms of correlation and agreement.

## 2. Materials and Methods

### 2.1. Study Design

A cross-sectional study with a single session to validate the POF sensor for monitoring the angle of flexion in the lower back was conducted at the School of Allied Health, University of Limerick, Ireland, between November 2017 and February 2018. The study was approved by the Faculty of Science and Engineering Ethics Committee, University of Limerick (Ref 2016_12_01).

### 2.2. Participants

Participants were recruited within the university community. The sample size was calculated based on one-sample correlation test. The null correlation (r_0_) was set to be 0 with significant level (α) of 0.05 and the power was selected to be 80%. A total of 18 participants (13 women and 5 men) were recruited. Hence, the estimated target correlation for the one-sample correlation test (r_a_) was 0.619. The inclusion criteria of this study were (i) adults from 18 to 59 years old, (ii) no specific low back pain (LBP) suffered within the previous six months and (iii) the ability to provide written informed consent. The exclusion criteria included (i) participants with history of serious back pain (scale > 5/10) and (ii) pregnant women. The baseline characteristics of the participants were specified by mean (± standard deviation) in which the age was 31.06 (9.85) years, height was 166 (8.44) cm, body mass was 65.72 (13.35) kg and body mass index (BMI) was 23.66 (3.64) kg/m^2^.

### 2.3. Instrumentation

**Figure 1:**
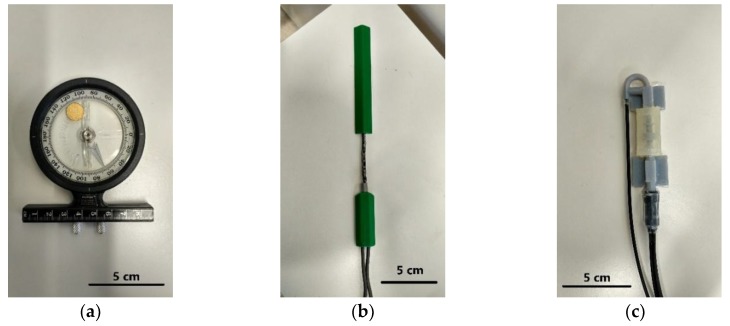
(**a**) Inclinometer. (**b**) Biometrics goniometer. (**c**) Plastic optical fiber (POF) sensor.

Lower back or lumbar (L1–L5) movement was measured using two sensors which were the Biometrics DataLink Data Acquisition System and the POF sensor of this investigation. In addition, an adjustable inclinometer as shown in Figure 1a was used as a baseline angle of movement in the trials.

#### 2.3.1. Biometrics DataLink Data Acquisition System

Biometrics DataLink Data Acquisition System, SG150/B (Biometrics Ltd., Newport, UK), was used in conjunction with a twin-axis electrogoniometer which is able to measure the bending angle simultaneously with the POF sensor, as shown in Figure 1b. The twin-axis Biometrics goniometer consists of two separate output connectors to measure the angles of movement in the sagittal (flexion/extension) and lateral (left/right) directions. It can measure the lumbar angle in a range of ± 150° with an accuracy of ±2°. The data obtained from the Biometrics goniometer were compared with the measurement from the POF sensor in the validation analysis.

#### 2.3.2. POF Sensor

The POF sensor in this investigation has been designed to measure the angle of the lumbar spine at L3L4 (The 2 vertebrae numbered L3 and L4 of the low back region). The sensor has been fabricated using 3-D printing and includes a plastic optical fiber which allows the tracking of curvature progression, as shown in Figure 1c. It works based on the change in optical intensity coupling between an input fiber coupled to three receiving fibers that are closely grouped together. The bend of the POF sensor causes an alignment mismatch between the input and output fibers and hence, power intensity loss from the angular misalignment between input and output tubes varies with bending angle. Through measuring the change of the coupled optical intensity ratio between three output fibers, the POF sensor can be used to measure the angle of the low back movement in both sagittal and lateral directions [20]. The POF sensor was calibrated using an optical setup system comprising a precise rotational stage, as shown in Figure 1, prior to being tested on human subjects. The output fiber’s intensity ratio was recorded in near real-time during the sensor characterization while rotating the stage at a different bending angle. The sensor exhibited an operating range of ± 12° for both sagittal and lateral bending with an average accuracy of 0.33°.

### 2.4. Procedure

A total of 18 volunteers with no specific back pain were selected according to demographic data including age, gender, weight and height. For measuring flexion, extension and lateral bend in both directions, participants were asked to stand up comfortably with their feet flat on the floor. The inclinometer, which is considered a suitable reference standard for measurement of lumbar flexion, was used as baseline angle movement. The spine is divided into 3 parts which are cervical, thoracic and lumbar regions. The cervical (neck) region consists of 7 vertebrae referred as C1 to C7. The thoracic (midback) region consists of 12 vertebrae numbered T1 to T12. The lumbar (lower back) region consists of 5 vertebrae which are numbered L1 to L5.

**Figure 2:**
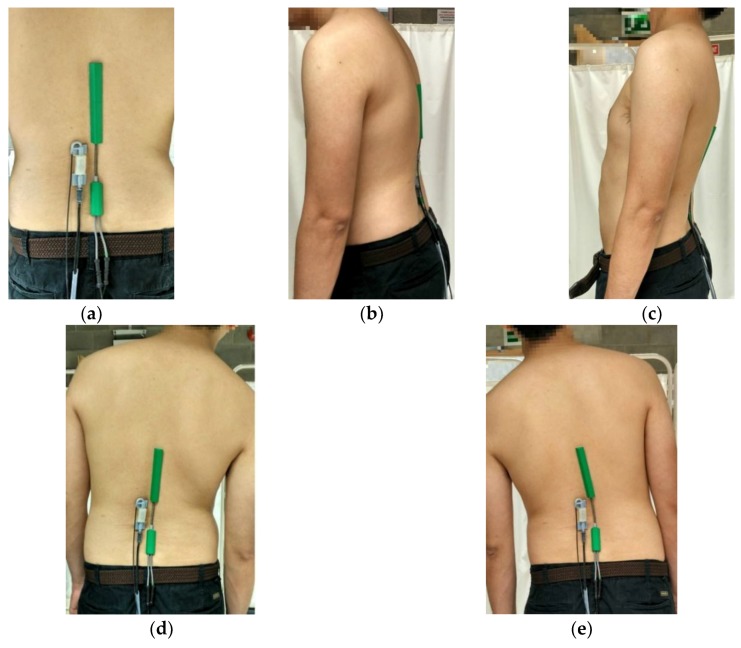
(**a**) Participant in a reference standing position having the POF sensor and Biometrics goniometer firmly attached next to each other on the skin at the low back-location. (**b**) Participant in the position for measuring flexion angle. (**c**) Participant in the position for measuring extension angle. (**d**) Participant in the position for measuring lateral bend (right) angle. (**e**) Participant in the position for measuring lateral bend (left) angle.

To measure the lumbar flexion from various subjects, the inclinometer was temporarily placed over the spinous process of T1T2 (the first two vertebrae of the midback located next to the neck) and T12L1 (the last vertebra of the midback and the first vertebra of the low back) of each person. The two inclinometers were aligned and set to 0° which was used as a reference position for each participant. The spinous process of T1T2 and T12L1 of each volunteer were indicated by an author who was a physiotherapist familiar with identification of spinal landmarks. The SG150/B Biometrics goniometer sensors with the length of 269 mm was located between L1 and L5 (the whole vertebrae column in the low back region) on the back of each patient which was identified using a manual palpation method. The 70 mm-long POF sensor was placed next to the Biometrics goniometer at the L3L4 section (the 2 vertebrae numbered L3 and L4 of the low back region) as shown in Figure 2a. 

Initially, the participants were asked to bend forward to 10° according to the reading from the inclinometer and hold for 10 s in order to measure the steady state flexion angle as shown in Figure 2b. Following the 10° bending forward, the participants were asked to stand up comfortably and bend forward again with a bending angle of 20° referenced from inclinometer and again requested to hold for 10 s. For measuring the extension, the participants were asked to bend backward until the reading of inclinometer was 10° as shown in Figure 2c. This posture was held for 10 s. After that, the participants were asked to bend more to the back until reaching 20° or at their maximum effort if they could not reach a 20° backward bending posture and hold for 10 s.

For lateral bend, the participants were asked to bend to the right for 10° and hold for 10 s as shown in Figure 2d before moving back to the reference position and then to the left for another 10° with 10 s holding as shown in Figure 2e. The angles of bending were indicated by the inclinometer. After that, a 20° lateral bending posture was measured for movement in both the right and left directions. Each position was held for 10 s to obtain a stable and reliable measurement. Every measurement was repeated three times to check for repeatability. The flexion angles of both the Biometrics goniometer and POF sensor were recorded together and simultaneously throughout the whole measurement process. Following the measurements, participant comfort was surveyed by asking the participant to provide a rating from 0–5, in which 0 represented no discomfort and 5 extreme discomfort.

### 2.5. Statistical Analysis

Data were statistically analyzed using the STATA version 14.0 software package (STATA Corp., College Station, Texas, USA). The data obtained from the measurement was considered to represent range of motion (ROM), which is used to indicate full movement of joint angles, in the low back (lumbar spine). A Shapiro-Wilk test was used to test the normality of the data in which normally distributed data are the null hypothesis. The data were found to have a *p*-value more than 0.05 for each bending position which means the null hypothesis cannot be rejected. Hence, the data distribution was normal. The POF sensor was investigated for its reliability using an intraclass correlation coefficient (ICC). The intraclass correlation coefficient is the medical measure of the reliability of measurements [21]. It shows the variation of the data measured by each sensor across three trials. There are different types of ICC depending on the measurement. In this work, the two-way mixed average score ICC was considered as the reliability of each sensor was studied. A scale for interpretation of the ICC was obtained from Cicchetti [22] in which ICC < 0.4 was poor, 0.4 < ICC < 0.59 was fair, 0.6 < ICC < 0.74 was good and ICC > 0.75 was excellent. The resolution of the instrument was evaluated using standard error of measurement (SEM) which can be calculated using standard deviation* sqrt(1-ICC) [23]. The minimal detectable change (MDC) was calculated based on SEM. Considering a 95% confidence interval, the MDC_95_ was calculated from sqrt(2)*1.96*SEM [24].

Validation of the POF sensor was studied using the Biometrics goniometer instrument together with the Pearson correlation coefficient (*r*-value) and 95% limits of agreement (LOA) Bland–Altman plots. The Bland and Altman plot is a commonly used statistical measure in medical applications. It is usually used to study the agreement of two different measurement techniques of the same measurand which were the Biometrics goniometer and POF sensor in this case [25]. The interpretation of the Pearson correlation was categorized as 0.00 to 0.29 being negligible correlation, 0.30 to 0.49 being low correlation, 0.50 to 0.69 being moderate correlation, 0.70 to 0.89 being high correlation and 0.90 to 1.00 being very high correlation [26]. The Bland–Altman plot was used to analyze the agreement between the measurements of the Biometrics goniometer and POF sensor from the mean difference and limits of agreement. In this work, the 95% LOA was considered a mean difference. All statistical results were rounded to two decimal places.

## 3. Results and Discussion

This study investigated the validity of the novel POF sensor by comparing the measurements of flexion, extension and lateral bend in both directions with a commercial Biometrics goniometer instrument. The mean angles and their standard deviation (SD) of the four lumbar movements including extension, flexion, lateral bend (left) and lateral bend (right) are shown in Table 1.

The mean values of the flexion angles, including flexion, extension and lateral bend, of both the POF sensor and the Biometrics goniometer exhibited significant statistical difference as the POF sensor measured the angle of the lumbar spine L3L4, whereas the biometrics goniometer measured the angle of lumbar L1L5. However, the mean values of both sensors showed a similar trend as they were increased to almost double when the flexion angles were doubled. The mean values of the POF sensor were consistently lower (0.52°–1.90°) compared to the mean values of the Biometrics goniometer (6.06°–14.34°). For the lumbar L1L5, the angle varied from 15° to 78°. The angle of the lumbar spine L3L4 varied in a range between 2° and 20° [27]. Note that the minus sign in this work represented extension and left flexion movements.

**Figure 3:**
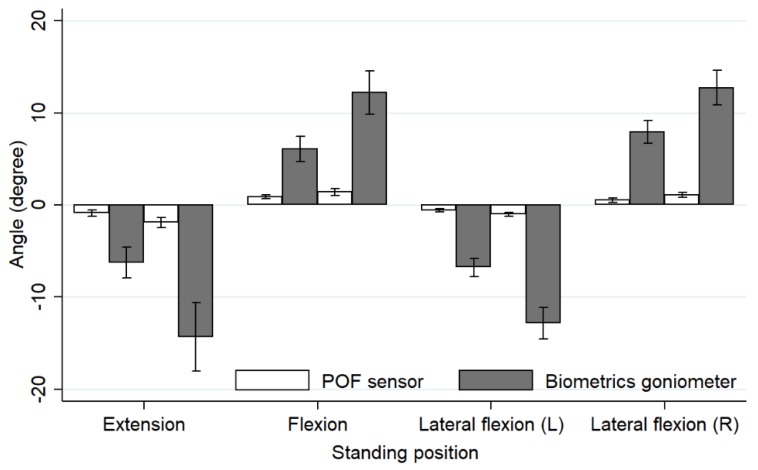
Bar chart showing mean angles and standard deviations of flexion, extension and lateral bend of both directions for the POF sensor (white bar chart) and Biometrics goniometer (grey bar chart).

The bar chart displaying a graphical comparison of a mean angle obtained from the POF sensor and Biometrics goniometer for each movement is shown in Figure 3. The lengths of the sensors were likely the key factor resulting in the large difference in the measured flexion angle shown in the bar chart. The Biometrics goniometer instrument which is 269 mm long can measure the lumbar angle (L1L5) in a range of ±150°. However, the POF sensor which is only 70 mm in length can measure the lumbar angle (L3L4) in a range of ±12°.

The reliability of the POF sensor was studied from the intra-rater reliability data including the intraclass correlation coefficient (ICC) with 95% confidence interval, standard error of measurement (SEM) and minimum detectable change (MDC) at the 95% confidence interval is reported in Table 2.

The reliability of the POF sensor in this study was considered good as the ICC values were in the range of 0.61 to 0.82 in the intra-rater reliability analysis. For the Biometrics goniometer, the ICC values in the intra-reliability analysis were between 0.67 and 0.82 which was also considered to be good. The good reliability was achieved due to (i) the firm and accurate placement of the sensors on participant’s back, (ii) testing procedure being performed before the actual test for familiarity and (iii) three repetitions of each movement. Without the firm placement of the sensors, the received signal could be unstable and inaccurate.

The correlation analysis between the POF sensor and Biometrics goniometer was studied using the Pearson correlation for which the r values are presented in Table 3 including the coefficient of determination (r^2^).

Overall, the measurements from the POF sensor and Biometrics goniometer exhibited a high correlation according to Cicchetti [22], as the Pearson correlation coefficient (r) is within the range of 0.77 and 0.81. Hence, the POF sensor which measured the angle of lumbar spine at L3L4 could be referred to the angle of the lumbar L1L5 measured by the Biometrics goniometer. The result angles measured by the two sensors can be convertible.

**Figure 4:**
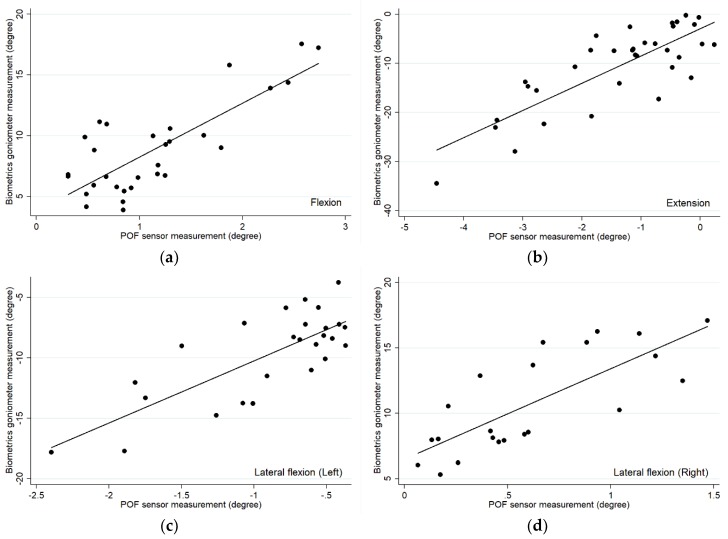
Scatter plots showing the linear relationship between the POF sensor and Biometrics goniometer for each movement including (**a**) flexion, (**b**) extension, (**c**) left lateral bend and (**d**) right lateral bend.

The relationships of the measurements between the POF sensor and Biometrics goniometer is demonstrated using a scatter plot as shown in Figure 4. The relationship between the angle values of these two measurements could be achieved from the scatter plot in Figure 4 in which the coefficients of determination were in the range between 0.59 and 0.65.

**Figure 5:**
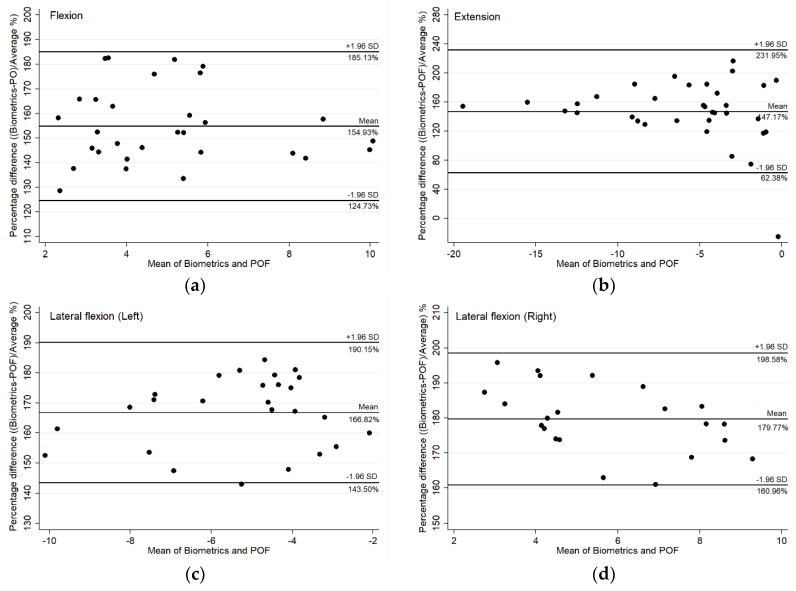
Bland–Altman plots between the POF sensor and Biometrics goniometer showing mean and percentage differences with the 95% limits of agreement (LOA) for each movement including (**a**) flexion, (**b**) extension, (**c**) left lateral bend and (**d**) right lateral bend.

In addition, the validity of the POF sensor was studied using Bland–Altman plots. The Bland–Altman plots with 95% confidence interval are shown in Figure 5. In this case, there is a proportional difference between the two measurements as the zero points were not included within the range of percentage differences between the POF sensor and Biometrics goniometer values. Therefore, the mean and percentage difference plots were considered instead of the absolute mean and difference plots.

From purely a consideration of the percentage difference in the Bland–Altman plots, a proportional difference existed between the measurements of the two sensors as different spine sections were measured. The proportional difference, which is a constant coefficient of variation, occurred because the POF sensor was developed to measure the flexion angle of the lumbar spine L3L4, which cover only the two vertebrae numbered L3 and L4 of the low back region, whereas the Biometrics goniometer was used to measure the lumbar curvature L1 to L5 which is the whole vertebrae column in the low back region. However, these two measurements showed a good correlation referring to the Pearson correlation coefficient [21], with r > 0.80 for flexion and extension and r > 0.70 for lateral bend both left and right.

The results exhibited a proportional difference variability between the measurements from the POF sensor and Biometrics goniometer as the two sensors were developed to measure different sections of the low back. However, due to the strong correlation, the POF sensor could be calibrated to measure the lumbar (L1L5) using the linear regression obtained from the scatter plots.

The main limitation for comparison within this investigation is that there was no compact commercial device available to measure the angle at the specific sections of spine, only for the whole upper or lower lumbar. It is not surprising that a sensor measuring ROM from L1L5 would display a greater magnitude of ROM than the POF sensor measuring ROM from L3L4. Hence, the percentage differences could reflect simply measuring different ROM, but importantly the correlations are strong. In addition, the relatively small sample size is a potential limitation in this study, as it was not based on the power calculation. However, it was considered to be consistent based on previous validation studies of similar devices [28].

## 4. Conclusions

The POF sensor demonstrated a strong correlation, and a proportional difference, with the biometrics goniometer across lumbar flexion, extension and lateral bend angles. The proportional difference in the Bland–Altman plots likely reflected the different sizes, and thus measurement regions, of the two sensors. The Biometrics goniometer was used to measure the flexion angle of the whole lumbar spine which is indicated from L1 to L5. However, the POF sensor, which has a smaller size, can be used to access the flexion angles with lower invasiveness in the low back region located at L3 and L4. The two sensors exhibited strong correlation when studied using the Pearson correlation technique. Therefore, the POF sensor can be reliably used to measure the flexion angle of the low back in a smaller region compared to the Biometrics goniometer. The result angles measured by the two sensors are convertible using linear regression analysis. The POF sensor could be of potential utility in measuring lumbar ROM in a manner which is minimally invasive, and where discrete sections of the spine are under specific investigation.

## Figures and Tables

**Table 1:** Mean angles (standard deviation) of the lumbar region measured by the POF sensor and Biometrics goniometer. The inclinometer was used as reference angles of 10° and 20°.

Device	Extension		Flexion		Lateral Bend (Left)		Lateral Bend (Right)	
	10°	20°	10°	20°	10°	20°	10°	20°
**POF sensor**	−0.90° (0.40°)	−1.90° (0.67°)	0.89° (0.23°)	1.41° (0.42°)	−0.58° (0.19°)	−1.02° (0.26°)	0.52° (0.28°)	1.13° (0.33°)
**Biometrics goniometer**	−6.28° (2.02°)	−14.34° (4.51°)	6.06° (1.65°)	12.21° (2.86°)	−6.79° (1.19°)	−12.82° (2.07°)	7.95° (1.49°)	12.73° (2.28°)

**Table 2:** Intra-rater reliability of the POF sensor and Biometrics goniometer.

Measurement	Intra-Rater Reliability	POF Sensor	Biometrics Goniometer
Extension	ICC(3,k) ^1,2^	0.61	0.74
	SEM ^3^	0.33	1.66
	MDC ^4^	0.93	4.61
Flexion	ICC(3,k)	0.63	0.67
	SEM	0.21	1.35
	MDC	0.57	3.75
Lateral bend (Left)	ICC(3,k)	0.66	0.71
	SEM	0.13	0.88
	MDC	0.36	2.43
Lateral bend (Right)	ICC(3,k)	0.82	0.82
	SEM	0.13	0.80
	MDC	0.36	2.22

^1^ ICC, intraclass correlation coefficient; ^2^ (3,k), two-way mixed average measures; ^3^ SEM, standard error of measurement; ^4^ MDC, minimum detectable change.

**Table 3:** Intra-rater reliability of the POF sensor and Biometrics goniometer.

ROM Measurement	Pearson Correlation Coefficient (r)	Coefficient of Determination (r^2^)
Extension	0.81	0.65
Flexion	0.80	0.64
Lateral bend (Left)	0.77	0.59
Lateral bend (Right)	0.77	0.59

## References

[1] Woolf A.D., Pfleger B. (2003). Burden of major musculoskeletal conditions. Bull. World Health Organ..

[2] Devereaux M.W. (2003). Neck and low back pain. Med. Clin. N. Am..

[3] Carroll L.J., Cassidy J.D., Côté P. (2004). Depression as a risk factor for onset of an episode of troublesome neck and low back pain. Pain.

[4] Bernard B.P., Putz-Anderson V. (1997). Musculoskeletal Disorders and Workplace Factors; A Critical Review of Epidemiologic Evidence for Work-Related Musculoskeletal Disorders of the Neck, Upper Extremity, and Low Back.

[5] Linton S.J. (2000). A review of psychological risk factors in back and neck pain. Spine.

[6] Dankaerts W., O’sullivan P., Burnett A., Straker L. (2006). Differences in sitting postures are associated with nonspecific chronic low back pain disorders when patients are subclassified. Spine.

[7] Campbell-Kyureghyan N., Jorgensen M., Burr D., Marras W. (2005). The prediction of lumbar spine geometry: Method development and validation. Clin. Biomech..

[8] Pearcy M., Whittle M. (1982). Movements of the lumbar spine measured by three-dimensional X-ray analysis. J. Biomed. Eng..

[9] Roudsari B., Jarvik J.G. (2010). Lumbar spine MRI for low back pain: Indications and yield. Am. J. Roentgenol..

[10] Roub L.W., Drayer B. (1979). Spinal computed tomography: Limitations and applications. Am. J. Roentgenol..

[11] Gehweiler J., Daffner R. (1983). Low back pain: The controversy of radiologic evaluation. Am. J. Roentgenol..

[12] Wong W.Y., Wong M.S. (2008). Detecting spinal posture change in sitting positions with tri-axial accelerometers. Gait Posture.

[13] Intolo P., Carman A.B., Milosavljevic S., Abbott J.H., Baxter G.D. (2010). The Spineangel^®^: Examining the validity and reliability of a novel clinical device for monitoring trunk motion. Man. Ther..

[14] O’Sullivan K., O’Sullivan L., Campbell A., O’Sullivan P., Dankaerts W. (2012). Towards monitoring lumbo-pelvic posture in real-life situations: Concurrent validity of a novel posture monitor and a traditional laboratory-based motion analysis system. Man. Ther..

[15] Mork P.J., Westgaard R.H. (2009). Back posture and low back muscle activity in female computer workers: A field study. Clin. Biomech..

[16] Lee R.Y., Laprade J., Fung E.H. (2003). A real-time gyroscopic system for three-dimensional measurement of lumbar spine motion. Med. Eng. Phys..

[17] Burdett R.G., Brown K.E., Fall M.P. (1986). Reliability and validity of four instruments for measuring lumbar spine and pelvic positions. Phys. Ther..

[18] Sheeran L., Sparkes V., Busse M., van Deursen R. (2010). Preliminary study: Reliability of the spinal wheel. A novel device to measure spinal postures applied to sitting and standing. Eur. Spine J..

[19] Mannion A.F., Knecht K., Balaban G., Dvorak J., Grob D. (2004). A new skin-surface device for measuring the curvature and global and segmental ranges of motion of the spine: Reliability of measurements and comparison with data reviewed from the literature. Eur. Spine J..

[20] Kam W., O’Sullivan K., O’Keeffe M., O’Keeffe S., Mohammed W.S., Lewis E. (2017). Low cost portable 3-D printed optical fiber sensor for real-time monitoring of lower back bending. Sens. Actuators A Phys..

[21] Shrout P.E., Fleiss J.L. (1979). Intraclass correlations: Uses in assessing rater reliability. Psychol. Bull..

[22] Cicchetti D.V. (1994). Guidelines, criteria, and rules of thumb for evaluating normed and standardized assessment instruments in psychology. Psychol. Assess..

[23] American Educational Research Association, American Psychological Association, National Council on Measurement in Educa (1999). Standards for Educational and Psychological Testing.

[24] Haley S.M., Fragala-Pinkham M.A. (2006). Interpreting change scores of tests and measures used in physical therapy. Phys. Ther..

[25] Altman D.G., Bland J.M. (1983). Measurement in medicine: The analysis of method comparison studies. J. R. Stat. Soc. Ser. D.

[26] Dennis E., Hinkle D., Wiersma W., Stephen G., Jurs S. (2003). Applied Statistics for the Behavioral Sciences.

[27] Damasceno L.H.F., Catarin S.R.G., Campos A.D., Defino H.L.A. (2006). Lumbar lordosis: A study of angle values and of vertebral bodies and intervertebral discs role. Acta Ortopédica Bras..

[28] O’Sullivan K., Verschueren S., Pans S., Smets D., Dekelver K., Dankaerts W. (2012). Validation of a novel spinal posture monitor: Comparison with digital videofluoroscopy. Eur. Spine J..

